# New Insights on the Palaeo-archaeological Potential of the Niokolo-Koba National Park, Senegal

**DOI:** 10.1007/s10437-023-09525-w

**Published:** 2023-04-15

**Authors:** Matar Ndiaye, Eric Huysecom, Katja Douze

**Affiliations:** 1 Institut Fondamental d’Afrique Noire Cheikh Anta Diop, University of Cheikh Anta Diop, BP: 206 Fann, Dakar, Senegal; 2 Laboratory of Archaeology of Africa and Anthropology, Section of Biology, University of Geneva, Quai Ernest-Ansermet 30, 1205 Geneva, Switzerland

**Keywords:** West Africa, Pleistocene, Early Holocene, Survey, Lithic industries

## Abstract

The study of the Palaeolithic in Senegal has made considerable progress in the last decade and has provided a renewed vision of the behavioral evolution of prehistoric populations in West Africa. The cultural trajectories within the region seem to be highly variable and bear witness to strong behavioral dynamics, the mechanisms of which still need to be better understood. However, the number of reliable, dated, and stratified sites, as well as the palaeoenvironmental data providing a context for populations in their palaeolandscapes, is still scarce. In order to provide new and solid data, we conducted new archaeological survey in the Niokolo-Koba National Park in south-central Senegal, aiming at a preliminary identification of Pleistocene and early Holocene sedimentary deposits. Here, we report an overview of the newly discovered industries found in different contexts. Most of the 27 identified sites show surface and out-of-context assemblages, but other sites are stratified and have all the criteria to justify the development of a long-term archaeological, geochronological, geomorphological, and palaeobotanical project. The Niokolo-Koba National Park, through which the Gambia River flows, is characterized by an abundance of sources of knappable material and by well-preserved sedimentary sequences. Therefore, archaeological research in the Niokolo-Koba National Park has the potential to provide major milestones in our understanding of the evolutionary dynamics at work in West Africa during the early periods of occupation of the region.

## Résumé

L’étude du Paléolithique au Sénégal connait des avancées considérables depuis cette dernière décennie, et permet d’apporter une vision renouvelée de l’évolution comportementale des populations préhistoriques en Afrique de l’Ouest. Les trajectoires culturelles au sein de la région semblent pourtant très variables et témoignent de fortes dynamiques comportementales, dont il s’agit à présent de mieux comprendre les mécanismes. Cependant, le nombre de sites fiables, datés et stratifiés, ainsi que les données paléoenvironnementales permettant de contextualiser les populations dans les paléopaysages, sont encore extrêmement rares. Dans l’optique d’apporter des nouvelles données solides, nous avons mené une première mission de prospections archéologiques dans le Parc National du Niokolo-Koba, dans le centre-sud du Sénégal, visant une reconnaissance des dépôts sédimentaires du Pléistocène et de l’Holocène ancien. Nous rapportons ici un aperçu des industries découvertes dans différents types de contextes. La majeure partie des 27 sites découverts montrent des assemblages en surface et hors contexte, mais d’autres sites sont stratifiés et possédent tous les critères justifiant le développement d’un projet archéologique, géochronologique, géomorphologique et paléobotanique à long terme. Le Parc National du Niokolo-Koba, traversé par le fleuve Gambie, est caractérise par l’abondance des matériaux taillables ainsi que par la très bonne préservation des séquences sédimentaires. Les recherches archéologiques dans le Parc National du Niokolo-Koba ont donc le potentiel d’apporter des jalons majeurs à notre compréhension des dynamiques évolutives à l’œuvre en Afrique de l’Ouest pendant les périodes d’occupation anciennes de la région.

## Introduction

The chrono-cultural sequence in Senegal, from the Pleistocene to the beginnings of the Holocene, has been a major research objective for the last 10 years in the study of the African Palaeolithic (e.g., Allsworth-Jones, 2021). Although there are still some gaps, the current evidence shows that West Africa follows an evolutionary dynamic that differs from other regions in the continent. This is consistent with the multi-regionalist cultural and behavioral trajectories for the period that saw the development of the first *Homo sapiens* in Africa (e.g., Scerri et al., 2018).

The Palaeolithic sequence of West Africa is characterized by strongly contrasting techno-cultural behaviors and rhythms of change, even within the same region, as in Senegal. Research carried out in the Falémé valley, in eastern Senegal, on remarkably well-preserved sedimentary sequences shows highly contrasted industries between the end of OIS 3 and the Early Holocene, between Middle Stone Age (MSA) and Later Stone Age (LSA) (Chevrier et al., 2018, 2020; Davidoux, 2021; Douze et al., 2021; Rasse et al., 2020; Schmid et al., 2022). On the contrary, renewed work on major sites such as Tiémassas near the Senegalese coast shows stability in MSA lithic industries over a long period, ranging from ca. 62 to ca. 25 ka (Niang & Ndiaye, 2016; Niang et al., 2018, 2020), which contrasts with the diversified industries found at continental sites in Senegal and Mali over the same period (Chevrier et al., 2016; Soriano et al., 2010). These changes in industries during OIS 2 (Chevrier et al., 2018) also contrast with the persistence of MSA technical traditions into the Holocene at Ndiayène Pendao sites on the Senegal River (Scerri et al., 2016, 2017) and Saxomununya and Laminia in south-eastern Senegal (Scerri et al., 2021).

The impact of ecological diversity on Palaeolithic populations, as well as the accessibility and abundance of knappable raw materials, are among the important factors to be considered in order to understand contrasts in technical behavior in prehistory. Today, Senegal is covered by at least three bioclimatic zones ranging from the Guinean savanna in the south to the Sahelian zone in the north and surrounded in the west by 530 km of the Atlantic coastline with distinctive environmental characteristics. On the other hand, more than half of Senegal is covered by washed hydromorphic soils composed of laterites and sands. The latter contain very few knappable raw material sources, while some coastal localities and the Birimian Supergroup in eastern Senegal present numerous outcrops of good fracturing quality. These factors most probably played a role in the modalities and dynamics of occupation of the Senegalese palaeolandscape, as well as in the technical behavior of the Palaeolithic populations. In order to assess these factors, it is necessary to develop robust palaeoenvironmental frameworks for the Pleistocene and early Holocene based on well-dated in situ archaeological sites. However, the poor preservation of sedimentary archives remains a major problem in West Africa and constitutes an obstacle to studying the earliest occupation periods. Consequently, this explains the difficulties in understanding the early techno-cultural dynamics at work in the region (e.g., Cerasoni et al., 2022).

**Fig. 1:**
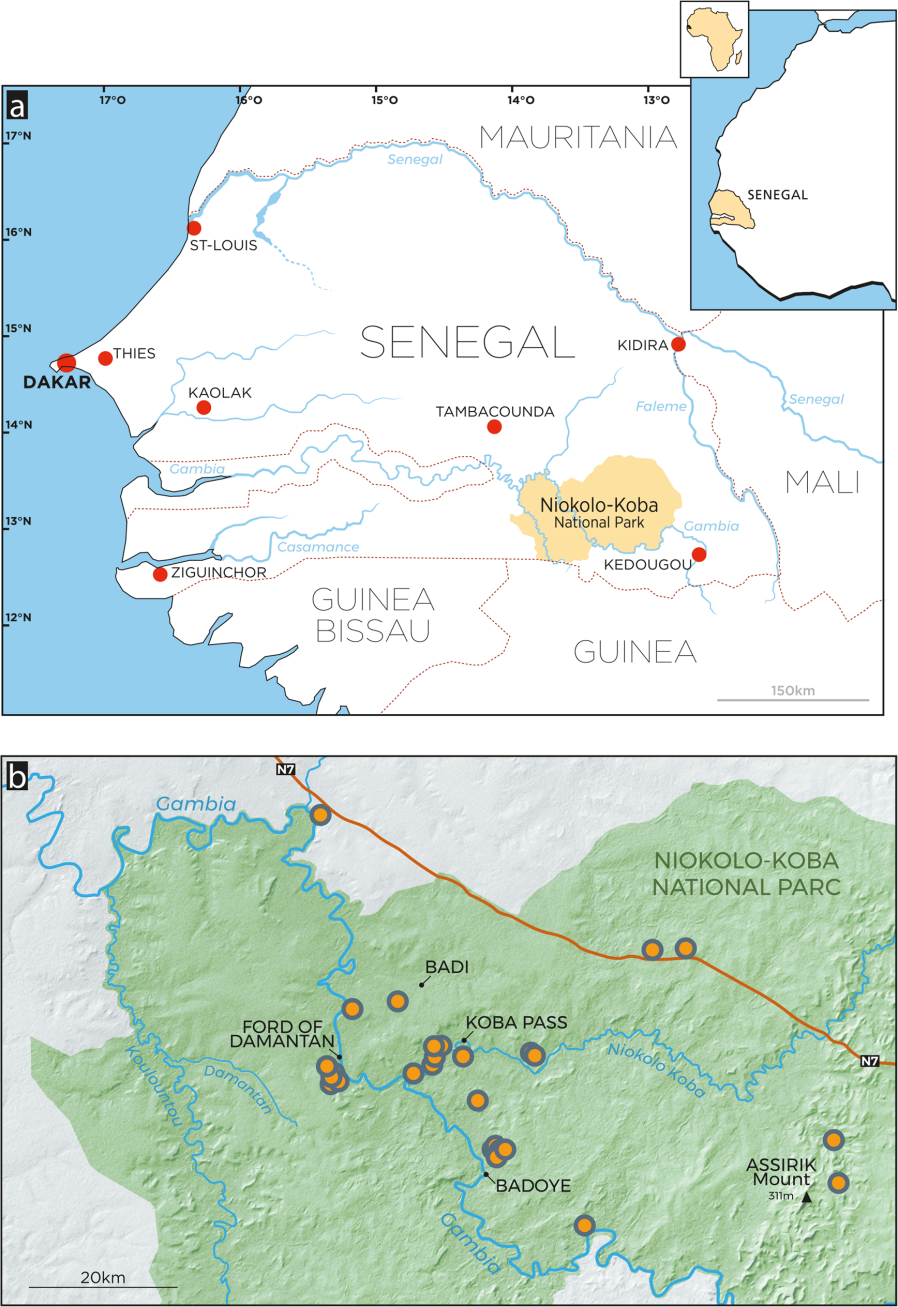
Location of the Niokolo-Koba National Park, Senegal: **a** Niokolo-Koba National Park in Senegal. **b** Dots showing the location of sites mentioned in the text. Artwork: D. Glauser

To begin to overcome some of these challenges, we conducted an archaeological survey in the Niokolo-Koba National Park (hereafter PNNK standing for *Parc National du Niokolo-Koba*) to establish an archaeological milestone in an intermediate zone between the Falémé valley in the far east of Senegal and the coastal zone in the west (Fig. 1). The Gambia River flows through the PNNK along a generally east–west axis, originating in the Fouta-Djalon, as does the Falémé river (Fig. 1a). The two basins present a geomorphological unity (Bassot, 1966) and cross the Birimian Supergroup, at least in part. Due to these points of convergence, and the very good preservation of the sedimentary deposits in the Falémé valley, the geo-archaeological potential of PNNK appeared significant. The aim was to identify the presence of Pleistocene and early Holocene sedimentary sequences containing buried and in situ archaeological materials using a multidisciplinary approach that includes archaeology, geomorphology, chronology, and palaeoenvironments. Here, we present the main results of Senegalese-Swiss archaeological and chronostratigraphic surveys in February 2022.

## History of the Park and Archaeological Work Between the 1960s and 1990s

The park was initially a hunting reserve (1926) and later a wildlife reserve (1953) before it was classified as a protected forest (1956). It was officially named “Niokolo-Koba National Park” on August 14, 1954, and established on 260,000 ha. The park grew exponentially in 1969 to reach its current surface of 913,000 ha. At the same time, the park’s inhabitants were pushed out of the classified area (e.g., Larrue, 2002; Majerovičová et al., 2022), thus limiting the anthropic impact on the fauna and flora. In 1981, the PNNK was inscribed on the UNESCO World Heritage List as a Biosphere Reserve, and since 2007, on the List of Endangered World Heritage Sites, due to mining, poaching, gold panning, river, and stream water pollution (https://whc.unesco.org/fr/list/153/documents/). The Direction des Parcs Nationaux of Senegal (DPN) is intensifying its commitment to the preservation of the biosphere, and numerous scientific research projects on fauna and flora are being conducted in the park, partly thanks to a partnership agreement signed between the DPN and the Institut Fondamental d'Afrique Noire of the Université Cheikh Anta Diop de Dakar (IFAN-UCAD) from which our mission also benefited.

The archaeology of the PNNK was explored from the late 1960s to the early 1980s. In 1969, Gaillard discovered knapped flakes between pebbles near the ford of Ba Foula Bé (also spelled Bafoulabé) (Descamps, 1971). Two consecutive missions in 1969, initiated by the University of Dakar, led to the collection of several hundred artifacts between Ba Foula Bé and the confluence of the Gambia and the Niokolo-Koba, as well as near the Koba Pass. Mainly made of jasper, sometimes of sandstone or quartz, these lithic artifacts, attributed to a “Levallois-Mousterian tradition,” have been thoroughly eroded by the river and were not found in sedimentary sequences (Descamps, 1971). In the alluvium of the Ba Foula Bé ford, a few less eroded artifacts are attributed to the Neolithic. A polished hematite axe was discovered in the vicinity of Badi, towards the north of the PNNK. Finally, Descamps mentions that the park does not belong to the Megalithic distribution zone and does not provide evidence for Iron Age and Protohistorical occupations.

In 1973, Michel (1973) published an important monograph on the geomorphological study of the Senegal and the Gambia river basins, which laid the foundation for a better understanding of the sedimentary and petrographic contexts of the PNNK. He also mentions the discovery of a small metabasite biface in fine alluvium near the Makana-Ko, a tributary on the right-hand side of the upper Niokolo-Koba. Michel, (1973) highlighted similarities between the sedimentary deposits of the Falémé and Gambia valleys, which led Camara & Duboscq (1983, 1984, 1990)—who used to work for IFAN carrying out major archaeological work on the Falémé valley—to concentrate on the Middle Gambia and the PNNK in the early 1980s (Camara & Duboscq, 1987a, b). Between 1982 and 1984, Descamps, Camara, and Duboscq undertook several campaigns in the PNNK and confirmed the similarities of the sedimentary deposits between the Falémé and Middle Gambia valleys. They also highlighted the archaeological significance of the PNNK with the discovery of several lithic industries. Some of these were part of Sarr’s master’s thesis on the lithic remains of Ba Foula Bé, Badoye, and Wouroli (Sarr, 1984).

The first industry, described as possibly “evolved Acheulean,” was discovered in Michel’s (1973) “low terrace," then renamed “middle alluvial level," most often corresponding to a gravelly or conglomerate crust (Camara & Duboscq, 1987b). It comprises 73 artifacts, showing a high percentage of side-scrapers, a few laminar pieces, end-scrapers, bifacially retouched tools, and a few polyhedral quartz tools. The industry was found in the marshlands of Badoye and on the site of Mbolor. According to Camara & Duboscq (1987b), understanding this industry requires additional research. 

A second industry, based on 101 pieces and qualified as "mousteroid," was not found in a stratigraphic context but appears among the rubble detached from bedrock outcrops, notably on a mound overlooking Badoye’s marshland. The pieces are mostly made of chert, or volcanic tuff for Badoye, and include many end-scrapers, notches, and denticulates.

The third industry was discovered in pebble banks, correlating with the dismantling of the “low alluvial level” or “underbank gravels” of Michel (1973). At the Badoye ford, it was discovered in an indurated level of pebbles and gravels trapped in the cracks of the bedrock outcrops. The industry was also collected at Ba Foula Bé, the ford of Pinassi (Niakassi), Wouroli, and the ford of Damantan. The industry is defined based on > 1000 pieces, mostly in chert with jasper facies. They are so strongly eroded that the knapping scars are sometimes almost erased. The characteristics show a dominance of Levallois debitage, micro-tools made on flakes or knapping waste, side-scrapers, notches, and denticulates. There are a few cores, pebble tools, bifaces and cleavers, and end-scrapers and drills. According to the authors, the industry could have different stratigraphic origins and represent a mixture of “evolved Acheulean” and “mousteroid” industries (Camara & Duboscq, 1987b).

A fourth industry, named “evolved Palaeolithic,” was recognized in the backfill formations that cover the alluvial levels. Found sporadically along the Niokolo-Koba and in the Mongo marshland, Camara and Duboscq discovered a stratified knapping workshop at the Badoye (or Impantyé) marshland, which led to an excavation campaign in early 1985. A total of 533 pieces were discovered on a surface of 10 m^2^, below 75–85 cm of sediments. The assemblage is composed of a large proportion of cores and is typologically dominated by core-scrapers. To our knowledge, no subsequent study has been published on this excavated assemblage.

Camara and Duboscq also suggested that the outcrops of the Birimian Supergroup rocks (Bassot, 1966; Michel, 1973) may have played an important role in the attractiveness of the PNNK to Palaeolithic populations, as is the case in the Falémé valley. A preliminary survey that we conducted in 2021 outside the PNNK, along the border of the Republic of the Gambia, in the vicinity of Tambacounda and the Vélingara crater, also led to this conclusion given the scarcity of Palaeolithic remains in these areas, even on the surface (Mayor et al., 2022). Our field observations concluded that the sedimentary sequences presented a greater depth of time as we approached the PNNK area, and the only industries found stratified during this mission were of MSA (lower level) and LSA (upper level) affinities, and were found near the Wassadou camp, on the northern border of the PNNK (Mayor et al., 2022).

## Survey Method

Our 2022 survey lasted for nine days and was carried out by a team of three archaeologists, two forest guards provided by the Direction des Parcs Nationaux of Senegal (DPN), and a driver. It aimed to: (1) make administrative and logistical contact with the DPN in Senegal; (2) assess the conditions for surveying in relation to the presence of wildlife, vegetation that has not been constrained since at least 1969, and the state of the roads; (3) to locate archaeological sites reported in previous work (Camara & Duboscq, 1987b; Descamps, 1971; Sarr, 1984); and (4) focus our research on areas of the park that have preserved Pleistocene and early Holocene sedimentary deposits containing Palaeolithic lithic industries.

The survey was therefore organized around targeted localities, identified from previous work, but also from topographic and hydrological network analysis based on IGN maps and *Bing* open-source hybrid aerial views*.* This preparation enabled us to target erosion and incision zones, set back from the immediate banks of the Gambia River covered by the most recent alluvial deposits, and from the tops of the lateritic hills (or bowé), which are often highly eroded, all according to the axes of circulation accessible by vehicle and on foot. The survey was not systematic and should therefore be seen as an initial assessment of the terrain in anticipation of large-scale surveys in the future. It covered an area from Badi in the north, Badoye in the south, the lower Niokolo Koba valley in the west, Damantan in the east, and a brief incursion into the vicinity of Mount Assirik, which is the highest point in the park at 311 m (Fig. 1b). The poor state of the trails, condition of our vehicle, bushfires caused by poachers which led to trees falling onto the tracks, and time constraints limited our progress beyond this area, which represents part of those already reported by previous work on the PNNK (Camara & Duboscq, 1987b; Descamps, 1971).

**Fig. 2:**
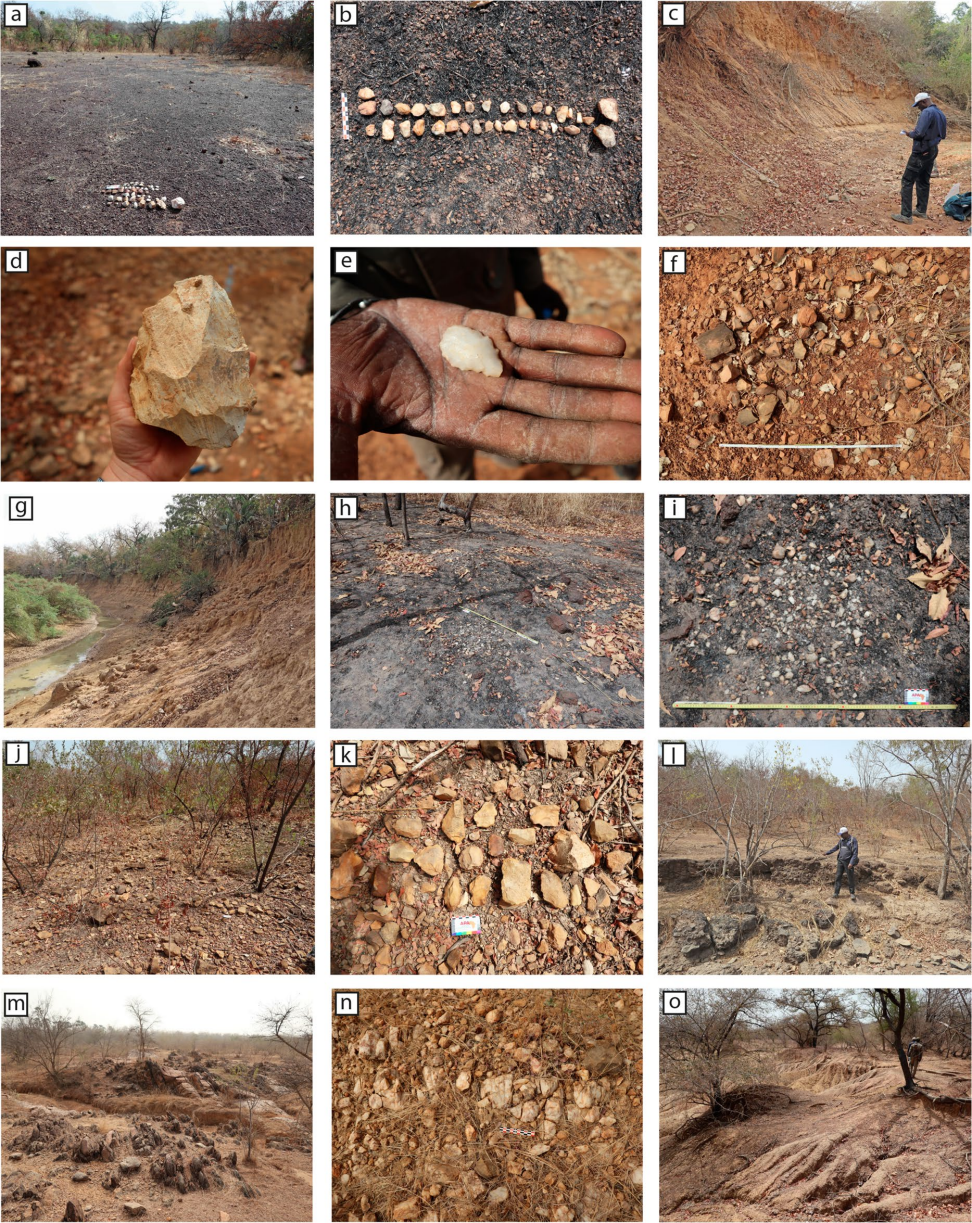
Different site contexts identified during the survey of the Niokolo-Koba National Park. **a** PNK19 site: surface on lateritic soils; **b** PNK20 site: surface lithics on lateritic soils; **c**, **d** PNK10 site: stratified lithics in sedimentary sequence; **e** PNK9 site: serrated point, surface find; **f** PNK9 site: knapping workshop cluster; **g** PNK21A site: sedimentary sequence; **h**, **i** PNK25 site: knapping workshop clusters; **j**, **k** PNK7 site: outcrop slopes with lithics; **l** PNK6 site: cemented pebbles and lithics bellow outcrop slopes; **m** greywacke or sandstone outcrops near Badoye; **n** quartz outcrops near Damantan; **o** PNK26, sedimentary sequence. Photos by the authors

**Fig. 3:**
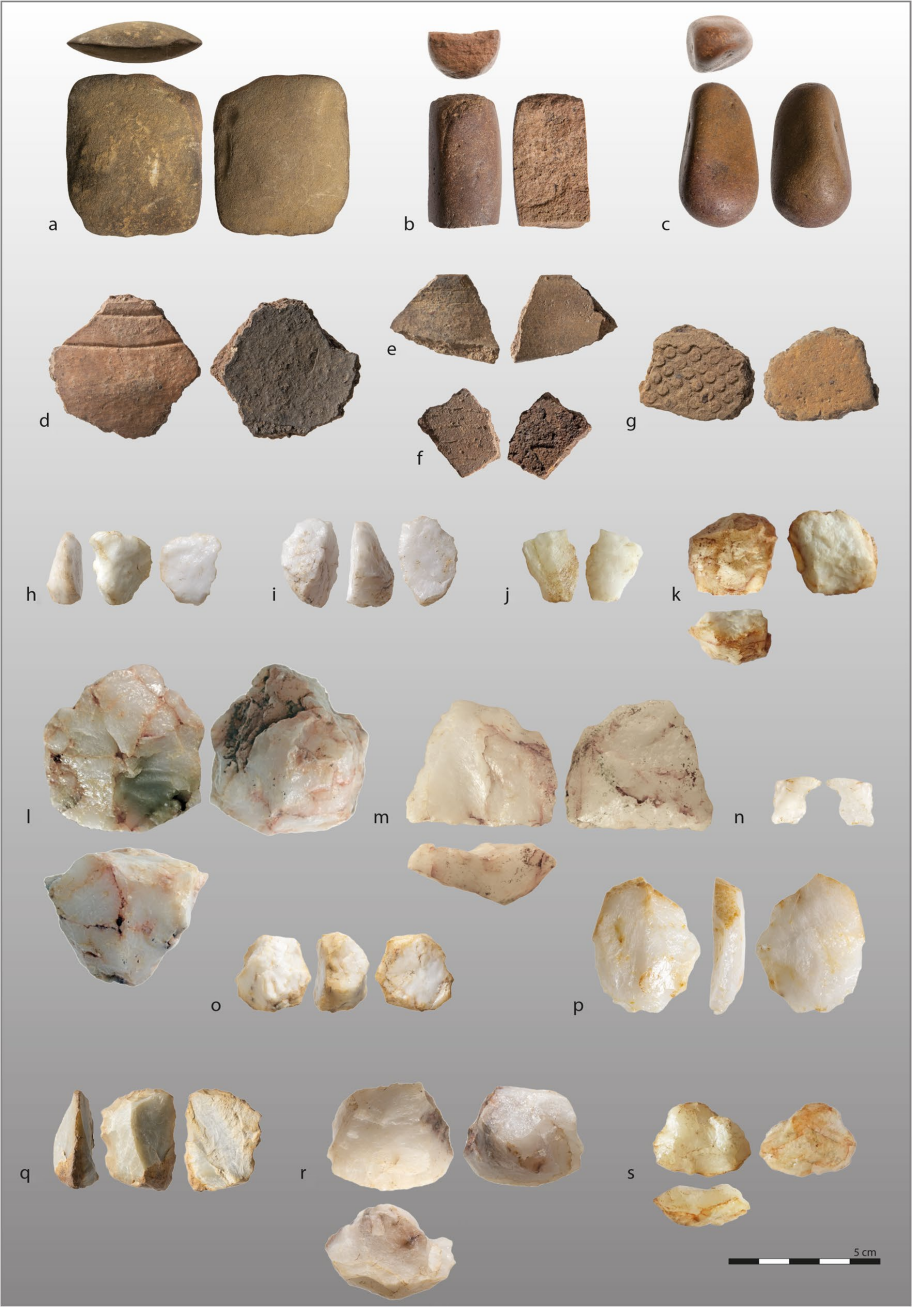
Examples of artifacts found in different sites of the Niokolo-Koba National Park. **a**–**f** (PNK13B): **a** polished axe, **b**–**c** hammerstones, **d**–**f** ceramic sherds; **g**–**i** (PNK19): **g** ceramic sherd, **h** notched flake, quartz, **i** end-scraper, quartz; **j**–**k** (PNK20): **j** broken blade-like flake, quartz, **k** bladelet core, quartz; **l** (PNK21B): centripetal flake core, quartz; **m**–**n** (PNK22): **m** planimetric flake core, quartz, **n** beak on flake, quartz; **o** (PNK18): thumbnail scraper-core, quartz; **p** (PNK26): quartz flake; **q**–**r** (PNK17): **q** end-scraper on flake, green chert, **r** elongated flake core, quartz; **s** (PNK13A): planimetric flake core, quartz. Photos: D. Glauser and K. Douze

**Fig. 4:**
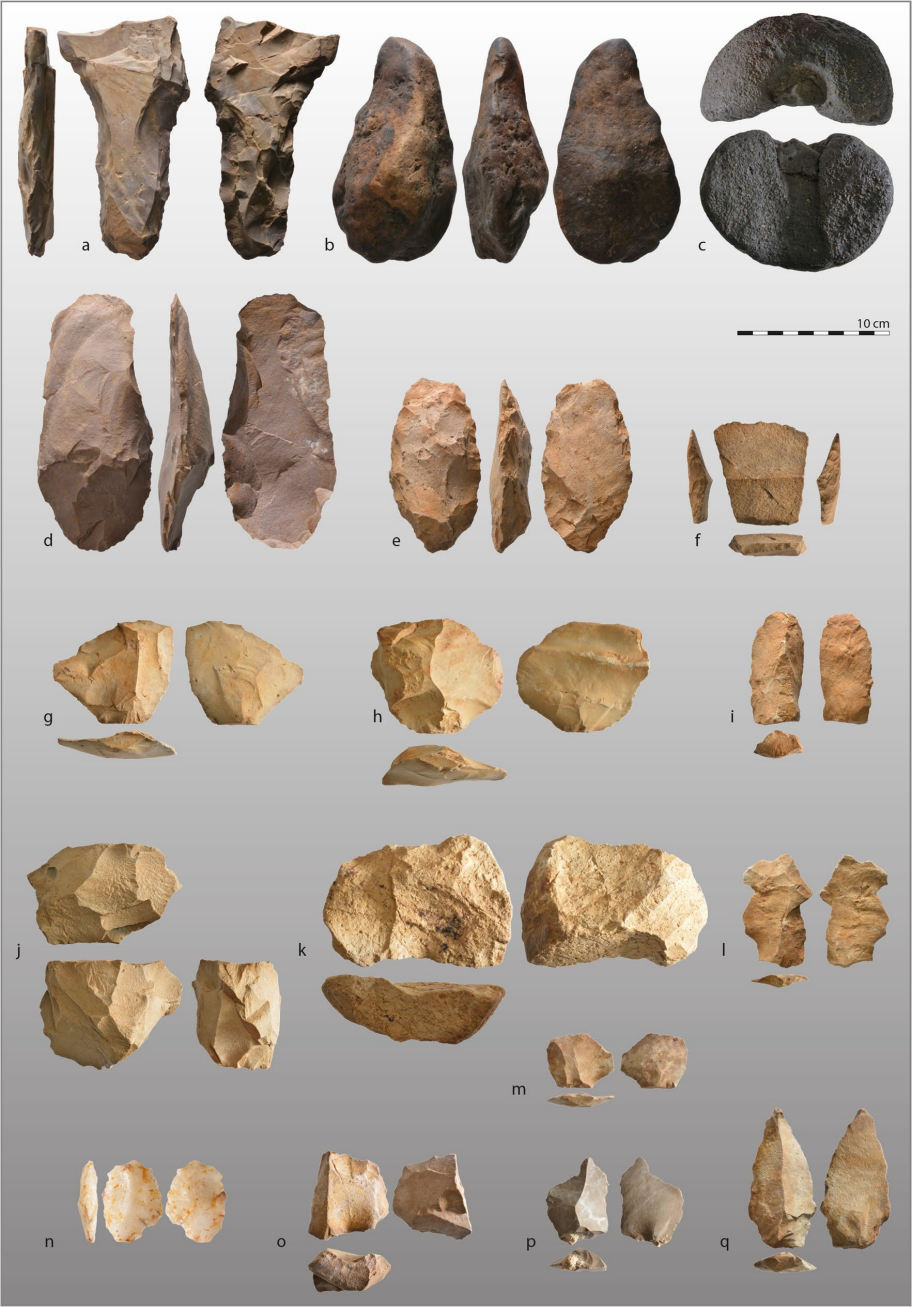
Examples of artifacts found in different sites of the Niokolo-Koba National Park (continued). **a**–**c** Isolated finds: **a** retouched tool, shaly sandstone, **b** biface, undetermined raw material, **c** Kwé, undetermined raw material; **d**–**f** (PNK9): **d** cleaver, greywacke, **e** partially bifacial tool, sandstone, **f** tranchet with backed edges, sandstone; **g**–**j** (PNK8): **g** convergent flake, sandstone, **h** flake, sandstone, **i** blade, sandstone, **j** multidirectional core, sandstone; **k**–**n** (PNK14): **k** levallois-like unidirectional core, sandstone; **l** blade-like flake, sandstone; **m** flake, sandstone, **n** flake, quartz; **o**–**q** (PNK10): **o** levallois-like preferential core, sandstone, **p** centripetal flake, greywacke, **q** elongated point, sandstone. Photos: K. Douze

The sites and places of interest were recorded on the free *Offline Maps* application for Android, which allows offline navigation and recording of GPS coordinates. Each point was associated with photographs of objects and context, and a description of the archaeological material and the sedimentary context represented at each marker. Only localities with a high density of materials over a limited area, corresponding globally to > 80 artifacts per 50 m^2^ in surface planimetry or to a well-defined archaeological unit in cross-section and dense in material, stratified in the sedimentary accumulations, were considered as sites (Figs. 1b and 2). Only a selection of about 40 highly diagnostic pieces, collected on the surface and destined to be washed away during the next rainy season, were selected for a more detailed description and illustration before being handed over to IFAN in Dakar (Figs. 3 and 4).

## Results of the Surveyed Sites

The survey mission carried out in 2022 led to the discovery of 25 archaeological sites, adding to the two sites discovered along the N7 road in 2021 (Fig. 1b; Mayor et al., 2022). Some of these sites are significant, exceptionally well-preserved sites that have been identified in sedimentary contexts that are favorable to the implementation of an archaeological research project involving cultural, geomorphological, geochronological, and palaeobotanical studies. Others, more numerous, are discoveries in surface spreading. Although informative, their potential needs more investigation, and their excavation is not prioritized in the framework of our future research program. Despite the absence of geomorphologists during this survey and the absence of archaeological excavations, we have identified different types of contexts with which industries are associated and which correspond to different preservation conditions of sites and artifacts (Fig. 2).

### Industries from Lateritic Crust Surface Contexts

This first context and the most frequent (*n* = 17 of 27 sites) was identified throughout the surveyed area. It consists of remains found in loose, sometimes sandy, ferruginous gravels spread over cemented lateritic crust terraces (Fig. 2a–b). In this context, the remains are always on the surface or slightly embedded in the crust (e.g., PNK19; PNK20, PNK22, PNK23) or have been brought to the surface by animal digging from the underlying pisolite sands (e.g., PNK12, PNK13A, PNK24). At some sites, the industries discovered in the crust were revealed by quarrying activities in the lateritic crust ahead of road construction (e.g., PNK1, PNK17).

In these contexts, the lithic artifacts have often been exposed to bushfires, leaving a greasy lens on their surface (e.g., Fig. 2b), but they bear otherwise very little signs of alteration. The almost exclusive use of quartz characterizes the industries. They all contain small quartz cores (ca. 2–5 cm maximum length) of unidirectional or multidirectional volumetric and unidirectional or centripetal planimetric exploitation to produce large flakes, blade-like flakes, and sometimes bladelets (Fig. 3k–m, r–s). Cores are always numerous. Typologically, a few retouched blade-like flakes or flakes are present and show a backed edge, a drill beak, isolated notches, and, more rarely, partial bifacial shaping as at PNK13A (Fig. 3h-i, n). Most often, these industries are found associated with a few isolated larger flakes in volcanic, siliceous, quartzite, or sandstone materials and may bear retouching, as with the scraper of green chert found at PNK19, which represents one of only two chert artifacts found during this survey (Fig. 3q). Some sites stand out for the presence of additional elements to this dominant assemblage composition. At PNK17, numerous quartz crystals were brought to the site, and although they do not appear to have been knapped, they sometimes bear small areas of battering that could be anthropogenic.

Sites PNK13B and PNK19 stand out by the presence of ceramic sherds, elongated hammerstones, and a small polished axe at PNK13B (Fig. 3a–g). The sherds at PNK13B have a grog temper and are quite thin (5–8 mm). They have a reddish-brown exterior, and a black interior with double or triple parallel incised decoration (Fig. 3d). One fragment of an everted rim is highlighted by an incised line, and another sherd shows a coarse white temper, possibly of sandstone. In PNK19, the ceramic sherd has a homogeneous paste with a coarse-grained hematite temper (1–6 mm) and shows a rolled cylinder impression decoration engraved with a large, dotted pattern (Fig. 3g). Another distinctive feature is the presence of small quartz thumbnail cores-scrapers at PNK19, also found in PNK18, although no ceramic sherds were identified in the latter (Fig. 3o). Finally, it should be noted that none of these sites shows backed segments, even in small quantities, as described at PNK2 discovered in 2021 in the same type of context, where large curved backed pieces on siliceous materials were identified within the matrix of scattered quartz artifacts (Mayor et al., 2022).

The quartz industries on the crust may suggest a late Palaeolithic period, probably a Holocene Later Stone Age, even in the absence of backed tools, or an early Neolithic period given the presence of a small polished axe. The shared characteristics identified for these assemblages from the top of the lateritic crust may be better distinguished based on a more detailed analysis of a larger corpus of finds. This distinction could help to assess whether the ceramics found at two of the 17 sites are associated with a quartz industry different from those at the other sites without ceramics. In their current state, these sites will not provide the chronostratigraphic and palaeoenvironmental information we seek. However, these industries may be preserved stratigraphically in as yet unexplored areas of PNNK, as is the case at the Wassadou 2 site on the northern border of the park (Mayor et al., 2022).

### Industries from Stratified Contexts in Sedimentary Sequences

The second context was frequently encountered along the Niokolo Koba river and towards Badoye. It includes lithic industries (*n* = 7 of 27 sites), sometimes found on several levels, in alluvial and colluvial-alluvial formations several meters deep (2–15 m depending on the site), incised by the current river channels (e.g., Fig. 2c, g). They present polyphasic sedimentary accumulations lying on the bedrock or on a cemented ferruginous bed of pebbles which could correspond to the level of “gravels under the bank," according to Michel, (1973). These accumulations are marked by alternations of more or less clayey and sandy silts of several shades (white-beige to red or mottled). They often show one or two levels of indurated channel floor gravels (10–50 cm thick), sometimes duplicated and layered, interbedded in the sequence. Most often, the sedimentary sequence is marked at the base by a unit of very indurated white-beige or white-yellow fine sediments (silts) with small scattered rounded pisolites. In contrast, the polyphasic fine sediments of the upper part of the sequence contain little to none. In these sequences, very well-preserved lithic industries (Fig. 2d) appear in artifact layers made visible by the gully-incisions. The artifacts lying on the slopes, eroded from the layers, attest to the archaeological richness of the sites. The sequence of several archaeological layers in the same locality (PNK9, PNK14, PNK16, and PNK21) allows us to assess the relative chronology of the techno-typological characteristics of the industries observed in PNNK.

There seems to be a correlation between the types of raw materials used in these industries and their stratigraphic position in these sequences. The industries included in the lower parts of the sedimentary sequences, found at PNK8, PNK9, PNK10, and PNK14, are made of quarzitic sandstone, silicified limestone, or greywacke, and occasionally of quartz. The core reduction sequences aimed to produce flakes, blade-like flakes, and triangular flakes of large dimensions, often > 8 cm in maximum length. The industries appear massive compared to the other PNNK sites (e.g., Fig. 4g–i, l–n, p–q). Some of the cores show planimetric exploitations, with uni- or bi-directional scar patterns and a geometric structure close to that of the Levallois conception (Fig. 4k, o). However, they show little investment in the preparation of the striking surface (i.e., little or no faceting) and the production surface (i.e., few scars of preparation and maintenance of the convexities). The platforms of the products, generally plain and thick, support this observation. At PNK8, PNK9, and PNK10, core-scrapers are also present, as well as multidirectional polyhedral cores, and show a non-Levallois core reduction concept for the production of flakes and elongated flakes (Fig. 4j). A few flakes are retouched on one or two edges and can be categorized as side-scrapers.

PNK9 is a sandstone or greywacke knapping workshop consisting of several large artifact clusters of ca. 2.5 m in diameter, visible on the surface (Fig. 2 f). However, some of these are still buried under 2 m of sediment. Near the clusters, a cleaver, a bifacial piece with an oblique distal edge, and a serrated unifacial point made on quartz as well as around 7-cm-long trapezoidal tranchet-axe with backed edges, made of coarse-grained sandstone, were found on the surface (Figs. 2e and 4d–f). The relationship between these tools and the knapping clusters remains to be clarified, mainly because of the unusual combination of these tool types in the Palaeolithic. At PNK9, PNK14, PNK16, and PNK21, the upper parts of the long sedimentary sequences show quartz-dominated industries composed of cores, flakes, and blade-like flakes quite similar at first sight to those identified on the surface in lateritic contexts.

To our knowledge, these rather massive sandstone or greywacke industries have no equivalent in Senegal, except for Camara & Duboscq’s, (1987b) mention of an “evolved Palaeolithic” found during unpublished excavations. Although it is tempting to link them to the MSA because of their lower stratigraphic position in the thick sedimentary sequences, their massive appearance, and the production of flakes, large blade-like flakes and points, we cannot exclude that they may bear witness to an as yet unknown LSA facies. The contexts in which these industries were discovered are, in any case, very favorable for geochronological and palaeoenvironmental studies. Thus, they represent a key research focus in implementing our large-scale project in the PNNK.

### The Knapping Clusters from the Decantation Basin

The context is that of a lowland, which could be likened to a decantation basin with temporary flooding, behind a bank bulge of the Gambia, surrounded by mounds of quartz outcrops. It is found on the left bank of the Gambia, past the Damantan ford. The discovery of an industry (*n* = 1 of 27 sites, PNK25) in this context is an isolated case, probably due to a lack of time for surveying the area (half a day). On the edge of this plain, a very large quartz block knapping workshop, in the form of several clusters, about 1.5 m in diameter, attests to an in situ occupation that is still partially buried but whose sedimentary context remains to be defined.

The massive quartz outcrops in the immediate vicinity served as a source for the knappers (Fig. 2n). The clusters are composed of important accumulations of angular debris, flakes, and cores that are often polyhedral, and imported blocks of quartz (Fig. 2h–i). The dimensions of the artifacts, alongside the fine fraction, are often large (5–12 cm maximum dimensions), probably due to the proximity of the primary quartz sources. The large polyhedral—or at least volumetric—ones, some planimetric cores, and the resulting large flakes, could reflect the industries of PNK21, PNK23, PNK24, and PNK26, which did not provide enough material for a precise characterization apart from these similarities with PNK25.

### Surface Industries from Outcrop Slopes

This context of discovery refers to eroded sandstone or greywacke outcrops on or around which artifacts and natural angular blocks are mixed (e.g., PNK7 and PNK11; Fig. 2j–k). This context was found in the direction of Badoye. The artifacts on the surface are often in good condition, with slightly blunted ridges and sometimes with surfaces torrefied by bushfire.

The industries are characterized by typical Levallois cores, flakes, and elongated flakes, often with faceted platforms, discoid artifacts, and one bifacial piece found at PNK7. The cores show numerous removals bearing witness to careful preparation of striking and exploitation surfaces. The remains are average size, rarely exceeding 8 cm in maximum length. Quartz is rarely used. The artifacts made of quarzitic sandstone, greywacke, and other materials (to be determined) were sourced by the outcropping rocks nearby. These technological characteristics remind us of the “mousteroid” identified by Camara & Duboscq (1987b) as well as the PNK1 site discovered in 2021, but which showed a dominant use of quartz, and the site of Wassadou 1, on the edge of the PNNK, which presented a stratified context (Mayor et al., 2022). This type of industry has never been found stratigraphically in the Falémé valley, but it has been found at Tiémassas (Niang & Ndiaye, 2016; Niang et al., 2018, 2020), in the Senegal River valley (Scerri et al., 2016, 2017), and at Laminia and Saxomununya (Scerri et al., 2021). Based on the ages available for these other contexts, this may be a late MSA, dating between the end of the Upper Pleistocene and the transition to the Holocene.

### Isolated Pieces Out of Context

Isolated pieces were identified, often very smoothed out, in coarse deposits at the bottom of present-day seasonal channels, on the bare substrate of these channels (Fig. 2m), and on the banks of the Gambia. These contexts have not been considered in the list of sites. Nevertheless, some of the collected pieces are significant from typological and chrono-cultural points of view. These include two small, highly smoothed bifaces found in seasonal gullies, a fragment of a “kwé,” and a large tool on a shaly sandstone slab retouched on the edges (Fig. 4a–c). The presence of a few bifaces attests to Acheulean occupations in the area, whose original sedimentary contexts are probably almost completely dismantled, as in the Falémé valley (Camara & Duboscq, 1983, 1984; Mayor et al., 2018, 2019, 2020, 2021, 2022). However, a good understanding of the dynamics at work and of the palaeolandscapes in the PNNK is necessary to better target the areas where these ancient palaeosoils are well preserved, which could lead to the discovery of in situ Acheulean industries.

## Conclusions

Non-systematic but targeted survey in the Niokolo-Koba National Park (PNNK) has made it possible to discover sites with strong chrono-cultural potential relating to the Upper Pleistocene and early Holocene. In general, Palaeolithic occupations in the PNNK are important, whereas they are barely visible for the Protohistoric period. The abundance and variety of knappable raw materials in the park have certainly played a role in this density of sites, as suggested by Camara and Duboscq in the 1980s. Petrographic studies are therefore essential for understanding the prehistoric occupation strategies in the PNNK landscapes. The long sedimentary sequences in which occupations are stratified provide optimal conditions for geomorphological, palaeoenvironmental, and geochronological studies. The importance of these stratified sites also lies in the fact that some industries, particularly those at the base of the sequences, have no equivalent in Senegal to our knowledge, thus providing new elements for understanding the cultural diversity of the West African Palaeolithic. Other contexts are less well-defined and need to be analyzed in more detail, including those that have yielded large quartz assemblages of LSA affinity spread out in lateritic contexts. These industries, coherent within each site, share similar technologies. However, they also seem to document different technical know-how from one site to another. Their study could help to refine our knowledge of the LSA facies of Senegal, which seem to be distinctive (e.g., Chevrier et al., 2020; Mayor et al., 2022). This survey has revealed the archaeological potential of PNNK and serves as a foundation for developing a large-scale Senegalese-Swiss archaeological and palaeoenvironmental research project in the near future.
